# Antioxidant, Anti-Inflammatory, and Anti-Diabetic Activity of Phenolic Acids Fractions Obtained from *Aerva lanata* (L.) Juss.

**DOI:** 10.3390/molecules26123486

**Published:** 2021-06-08

**Authors:** Aleksandra Pieczykolan, Wioleta Pietrzak, Urszula Gawlik-Dziki, Renata Nowak

**Affiliations:** 1 Chair and Department of Pharmaceutical Botany, Medical University, 1 Chodźki Street, 20-093 Lublin, Poland; aleksandraoleszek@umlub.pl (A.P.); wioletapietrzak@umlub.pl (W.P.); 2 Department of Biochemistry and Food Chemistry, University of Life Sciences, 8 Skromna Street, 20-704 Lublin, Poland; urszula.gawlik@up.lublin.pl

**Keywords:** *Aerva lanata* (L.) Juss., LC MS/MS, phenolic acids, lipoxygenase, xanthine oxidase, antioxidant, α-amylase, α-glucosidase

## Abstract

Many plants that are commonly used in folk medicine have multidirectional biological properties confirmed by scientific research. One of them is *Aerva lanata* (L.) Juss. (F. Amaranthaceae). It is widely used, but there are very few scientific data about its chemical composition and pharmacological activity. The aim of the present study was to investigate the chemical composition of phenolic acid (PA)-rich fractions isolated from methanolic extracts of *A. lanata* (L.) Juss. herb using the liquid/liquid extraction method and their potential antioxidant, anti-inflammatory, and anti-diabetic properties. The free PA fraction (FA), the PA fraction (FB) released after acid hydrolysis, and the PA fraction (FC) obtained after alkaline hydrolysis were analysed using liquid chromatography/electrospray ionization triple quadrupole mass spectrometry (LC-ESI-MS/MS). The phenolic profile of each sample showed a high concentration of PAs and their presence in *A. lanata* (L.) Juss. herb mainly in bound states. Thirteen compounds were detected and quantified in all samples, including some PAs that had not been previously detected in this plant species. Bioactivity assays of all fractions revealed high 2,2-diphenyl-1-picrylhydrazyl (DPPH^•^) (2.85 mM Trolox equivalents (TE)/g) and 2,2-azino-bis-3(ethylbenzthiazoline-6-sulphonic acid) (ABTS^•+^) (2.88 mM TE/g) scavenging activity. Fraction FB definitely exhibited not only the highest antiradical activity but also the strongest xanthine oxidase (XO) (EC_50_ = 1.77 mg/mL) and lipoxygenase (LOX)(EC_50_ = 1.88 mg/mL) inhibitory potential. The fraction had the best anti-diabetic properties, i.e., mild inhibition of α-amylase (EC_50_ = 7.46 mg/mL) and strong inhibition of α-glucosidase (EC_50_ = 0.30 mg/mL). The activities of all analysed samples were strongly related to the presence of PA compounds and the total PA content.

## 1. Introduction

Many contemporary medicines are produced from traditionally used plants; hence, increasing interest in ethnobotany has been observed. One of such species is *Aerva lanata* (L.) Juss. (*Amaranthaceae*), which is an important medicinal plant growing as a common weed in fields and wasteland in tropical regions of Africa and Asia [1]. It has been known under many names depending on the region of occurrence and has been shown to have multifarious applications in traditional medicine [2,3]. In Ayurveda, *A. lanata* (L.) Juss. is described as a diuretic with anti-inflammatory, antibacterial, anthelmintic, and analgesic effects and as an agent used in the treatment of diabetes [4]. Leaf and root paste has anti-inflammatory and antibacterial properties and is applied to treat pustules and skin infections [5,6]. It can also be an ingredient in cosmetic products.

Due to its popularity in folk medicine, the plant has been the subject of pharmacological and chemical studies for approximately 30 years. Its wide therapeutic activities are associated with the content of active compounds such as alkaloids [7,8], saponins, terpenoids, and various phenolic compounds [9,10,11,12]. Numerous recent studies confirm the wide application possibilities of *A. lanata* (L.) Juss. Many reports in the literature have demonstrated itsantioxidant [10,13,14], anti-cancer [15,16], anti-inflammatory [17,18], and hepatoprotective effects [15,19]. The plant is widely known for nephroprotective, diuretic, and antiurolithic activity, which havebeen confirmed by scientific research [17,20,21]. Additionally, the anti-diabetic potential of *A. lanata* (L.) Juss. root extracts related to inhibition of enzymes has been recently reported [22,23,24].

Diabetes is one of the 21st century’s “diseases of civilization”. It has been linked to oxidative stress, which arises mainly through oxidation, oxidative degradation of glycated proteins, and nonenzymatic protein glycation. Animpaired antioxidant defence mechanism provokes cellular and enzyme damage and lipid peroxidation, which subsequently lead to the development and progression of insulin resistance and hyperglycaemia [25,26]. The main ingredients of drugs used in diabetes treatment, such as acarbose, miglitol, and voglibose, exert their effects by inhibiting α-glucosidase and α-amylase. Hence, the constant interest of scientists in the search for these new natural inhibitors in plants is observed [27].

Polyphenols constituting a large group of naturally occurring molecules are well known not only for their antioxidant activity but also for their hypoglycaemic properties. For example, flavonoids and phenolic acids have been suggested to inhibit α-glucosidase and α-amylase to support the treatment of diabetes type 2 [28]. Most phenolic acids have been found to exhibit considerable clinical or in vivo anti-diabetic activity as well [29]. Additionally, one of the ways to prevent diabetes is the consumption of polyphenol-rich foods [30].

To the best of our knowledge, there are no quantitative and qualitative data about the presence of phenolic acids in *A. lanata* (L.) Juss. and their possible relationship with the antioxidant and anti-diabetic activity of extracts from the herb of this plant. Therefore, in this research study, phenolic acid-rich fractions were isolated, and their antioxidant activity and ability to inhibit α-glucosidase and α-amylase were examined. The formation of ROS (reactive oxygen species) is connected with lipoxygenase (LOX) and xanthine oxidase (XO) activity; hence, another aim of the study was to assess the activities of LOX and XO.

## 2. Results and Discussion

Phenolic acids are widely distributed among plants. For some years, they were regarded as metabolically inactive substances present in small amounts and having no role in the biological activity of plants. However, recent studies have changed this point of view. Currently, phenolic acids receive special attention due to their strong antioxidant activity. Recent pharmacological studies have shown a number of activities of phenolic acids, including anti-diabetic effects [29,31].

As mentioned before, *Aerva lanata* (L.) Juss. is widely used in traditional medicine due to its numerous health-promoting properties. The plant is included in the Russia State Pharmacopoeia as a diuretic medicine. The herb is recommended for internal administration as a decoction [32,33].

Additionally, there are reports of the anti-diabetic activity of methanol extracts of *A. lanata* (L.) Juss. roots. They suggest that alkaloids from methanol extracts are responsible for this action [34]. In turn, our results clearly indicate a potent α-glucosidase inhibitory effect of the phenolic acid fractions isolated from the herb of *A. lanata* (L.) Juss.

### 2.1. Analysis of the Phenolic Acid Profile in the Herb of Aerva lanata (L.) Juss.

Phenolic acids generally occur in plants in a free or bound form, e.g., as esters or glycosylated compounds. They have two main parent structures: hydroxycinnamic and hydroxybenzoic acids. Ferulic, caffeic, p-coumaric, and sinapic acids are the most abundant hydroxycinnamic acids. The most important hydroxybenzoic acids are gallic, vanillic, and syringic acids [35,36].

The isolation of plant secondary metabolites, such as phenolic acids, is a very important step in phytochemical analysis and phytotherapy research. Extraction of fractions containing complexes of compounds from a given group of metabolites facilitates assessment of their biological activity and the relationship of the plant material with the bioactivity.

In the present study, phenolic acids from *Aerva lanata* (L.) Juss. herb were isolated and quantified for the first time using a standard procedure, and the form of phenolic acids (free, bound) contained in the dry material was determined. The liquid/liquid extraction (LLE) and solid phase extraction (SPE) methods were used for isolation and purification of fractions of free phenolic acids (FA) and acids liberated after acidic (FB) and alkaline (FC) hydrolysis. A rich qualitative composition of phenolic acids and a higher concentration of phenolic acids (over 1000 times higher) were observed in the isolated fractions compared with the plant material. Phenolic acids were present in *A. lanata* (L.) Juss. in both the free and bound states (see Supplementary Materials).The compounds were identified, and their content was determined with the LC MS/MS method in the MRM mode.

**Figure 1:**
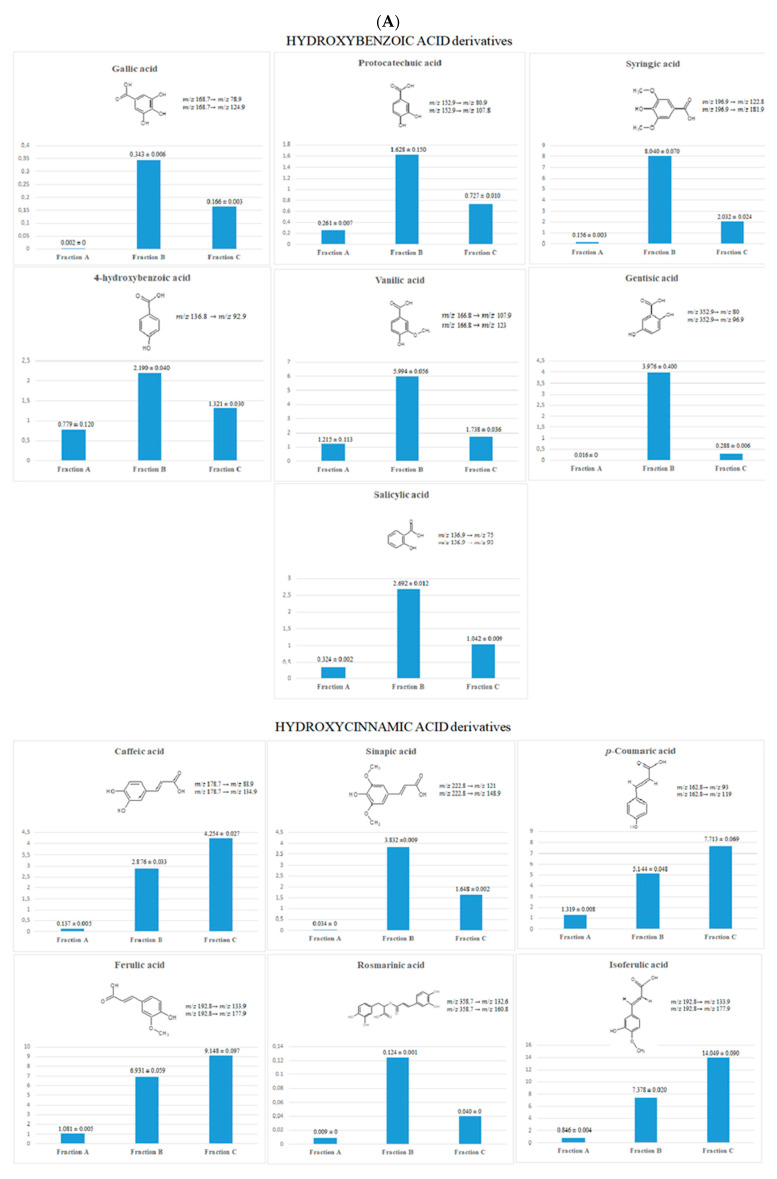

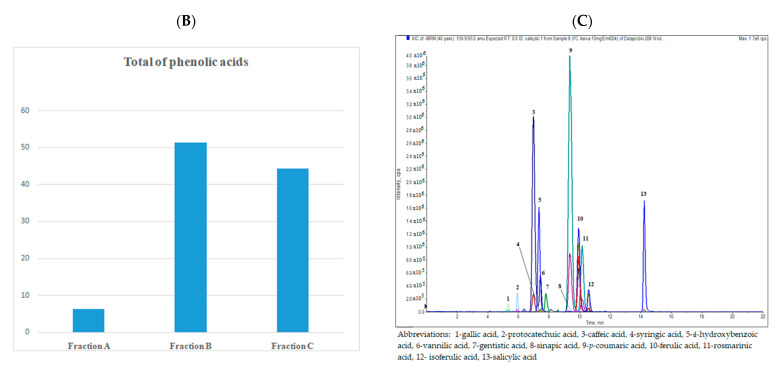
Phenolic acid profile in the herb of *Aerva lanata* (L.) Juss. (µg per g dry plant material)—LC-ESI-MS/MS results. (**A**) Comparison of the content of phenolic acids in the analysed fractions; (**B**) total phenolic acid content in fractions FA, FB, FC; (**C**) LC-ESI-MS/MS chromatogram of phenolic acids detected in the FB sample obtained in the MRM mode. Abbreviations: FA, fraction of free phenolic acids; FB, fraction of phenolic acids obtained after acidic hydrolysis; FC, fraction of phenolic acids obtained after alkaline hydrolysis.

A comparison of the phenolic acid profile in the dry plant material is shown in Figure 1. The fraction of free phenolic acids (FA) contained the lowest amounts of these compounds. The total content of free phenolic acids was 6.199 µg per g of dry plant material. The results of the FA analysis show the highest content (about 40%) of p-coumaric acid (1.319 μg ± 0.008 per g of dry plant material) and vanillic acid (1.215 μg ± 0.113 per g of dry plant material).

In acidic and alkaline hydrolysis, glycosidic and ester bonds are broken, and the respective forms are released into free phenolic acids [37]. Fraction B (FB) contained very large amounts of phenolic acids liberated after acidic hydrolysis (the total content of PA was 51.335 µg per g dry plant material, which was about 9 times higher than in fraction A). The main compounds in FB were syringic acid (8.040 μg ± 0.070 per g of dry plant material) and isoferulic acid (7.378 μg ± 0.020 per g of dry plant material).

Fraction C (FC) contained phenolic acids released from the water fraction by alkaline hydrolysis. The total content of phenolic acids was 44.375 µg per g of dry plant material, which was approximately a 7 times higher level than in fraction A. The results showed the largest amount of isoferulic and ferulic acids (14.049 μg ± 0.090 and 9.148 μg ± 0.097 per g of dry plant material, respectively).

The content of PAs in the dry plant material and their occurrence in both free and bound forms was determined for the first time. Fraction A contained negligible amounts of PA, compared with fractions FB and FC. Fraction B contained phenolic acids that were also detected in FC; however, in most cases, syringic, vanillic, gentisic, sinapic, 4-hydroxybenzoic, salicylic, and protocatechuic acids (mainly hydroxybenzoic acid derivatives, except synapic acid) were present in a larger amount than in FC. The largest differences between fractions B and C were noted in the content of gentistic, vanillic, and syringic acids. In comparison with acidic hydrolysis, alkaline hydrolysis released the highest amount of hydroxycinnamic acids, especially ferulic acid, isoferulic acid, caffeic acid, and p-coumaric acid. The acidic hydrolysis largely increased the content of phenolic acids.

As mentioned earlier, the extraction procedure was very efficient and there was an increase in the concentration of phenolic acids (up to 1000 times) in the obtained fractions, compared with the concentration of the phenolic acids in the dry plant material. Table 1 shows the content of phenolic acids in the isolated fractions. In our study, a high concentration of isoferulic, ferulic, and p-coumaric acids was observed in all dry fractions.

To the best of our knowledge, this is the first qualitative and quantitative analysis of the phenolic acid content in *Aerva lanata* (L.) Juss. conducted using the rapid, sensitive, and effective liquid chromatography-electrospray ionization-tandem mass spectrometry method (LC-ESI-MS/MS). Although there are data from several previous studies in the literature in this field, the data are incomplete and should be confirmed. In 1991, Zapesochnaya et al. reported isolation of syringic and vanillic acids [12]. The tests carried out by Mammen et al. showed the presence of some phenolic acids by means of paper chromatography (PC) and thin layer-chromatography (TLC) methods [9,38]. The presence of ferulic, gallic, and ellagic acids was also reported by other researchers [10,39,40]. Recently, the presence of cinnamic and chlorogenic acids has been demonstrated too [41].

In this study, a comprehensive LC-ESI-MS/MS analysis of the content of phenolic acids and their form in *A. lanata* (L.) Juss. was performed for the first time. Moreover, there are no studies determining free phenolic acids and phenolic acids released after acidic and alkaline hydrolysis from this plant.

### 2.2. Analysis of Antioxidant and Anti-Inflammatory Activity

The antioxidant (DPPH^•^ and ABTS^•+^) and anti-inflammatory (XO, LOX) activities of the crude methanol extract and dry PA fractions obtained from *Aerva lanata* (L.) Juss. were determined, and the results of this research are shown in Table 2.

Among the fractions, the highest antioxidant capacities in the DPPH^•^ and ABTS^•+^ assay were detected for fraction B: 2.85 mM Trolox/g dry weight of fraction and 2.86 mM Trolox/g dry weight of fraction, respectively. In turn, fraction A exhibited the lowest results (0.49 mM Trolox/g dry weight of fraction for DPPH^•^ and 0.57 mMTrolox/g dry weight of fraction for ABTS^•+^). Compared with the fractions, the crude methanol extract had lower antioxidant activity (0.29 mM and 0.22 mM Trolox/g dry extract for DPPH^•^ and ABTS^•+^, respectively). Fractions FB and FC displayed definitely higher DPPH^•^ free radical scavenging activity relative to free phenolic acid fraction FA.

Additionally, the effect of the crude methanol extract and fractions obtained from the *A. lanata* (L.) Juss. herb in an in vitro XO- and LOX-mediated inflammatory assay was analysed (Table 2). Lipoxygenase (LOX) transforms linoleic, arachidonic, and other polyunsaturated fatty acids into biologically active metabolites implicated in inflammation [42]. In turn, xanthine oxidase (XO) catalyses hydroxylation of hypoxanthine into xanthine and, finally, xanthine into uric acid. Overproduction of uric acid or inadequate secretion thereof results inhyperuricemia and subsequently results in deposition in joints, leading in consequence to inflammation with pain [43].

As shown in the table, high LOX inhibitory potential was found for all samples. Fraction B displayed the highest values (EC_50_ = 1.77 mg/mL), whereas the lowest value was determined in the case of fraction A (EC_50_ = 4.16 mg/mL). In turn, the highest XO inhibitory potential was found for fraction B (EC_50_ = 1.88 mg/mL), and the lowest value was determined in the case of fraction C (EC_50_ = 4.15mg/mL). Overall, all samples exhibited high LOX and XO inhibitory activity, as they contain bioactive compounds that can be used in the treatment of LOX- and XO-induced diseases and inflammation. To the best of our knowledge, there are limited studies concerning the lipoxygenase and xanthine oxidase inhibitory activities of *A. lanata* (L.) Juss. As reported by Sutharson et al. [44], aqueous extracts from *A. lanata* (L.) Juss. obtained with the Soxhlet method showed considerable XO inhibitory activity (IC_50_ = 62.53 μg/mL).

The inhibition mode was investigated using the Lineweaver–Burk plot analysis. The enzyme activity was measured at various substrate concentrations in the presence of the inhibitor at different concentrations, which was used to construct the Lineweaver–Burk plot. Classification of the inhibition type as competitive, non-competitive, or uncompetitive was done on the basis of these plots [45].

**Figure 2:**
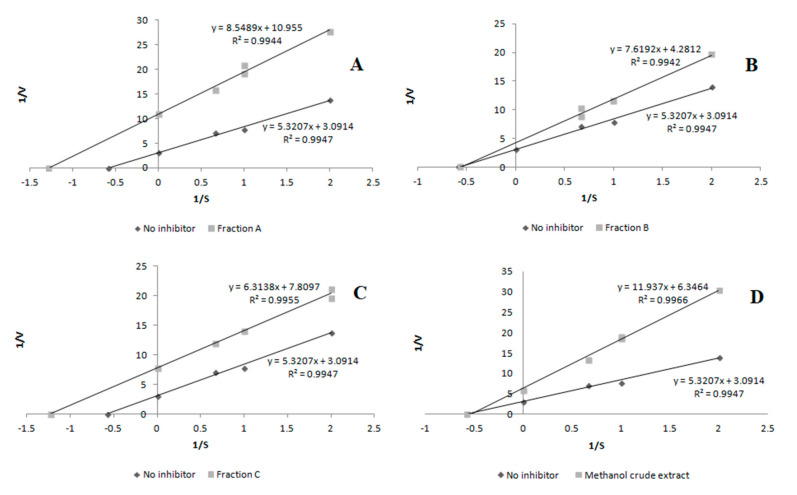
Mode of lipoxygenase (LOX) inhibition by samples from *Aerva lanata* (L.) Juss. herb. (**A**) Fraction A; (**B**) fraction B; (**C**) fraction C; (**D**) crude methanol extract. V, rate of enzymatic reaction expressed as the absorbance unit; S, substrate concentration.

In the case of lipoxygenase, the results reveal that fraction A and fraction C acted as uncompetitive inhibitors, while fraction B and the crude methanol extract demonstrated a non-competitive mode of inhibitory activity (Figure 2).

**Figure 3:**
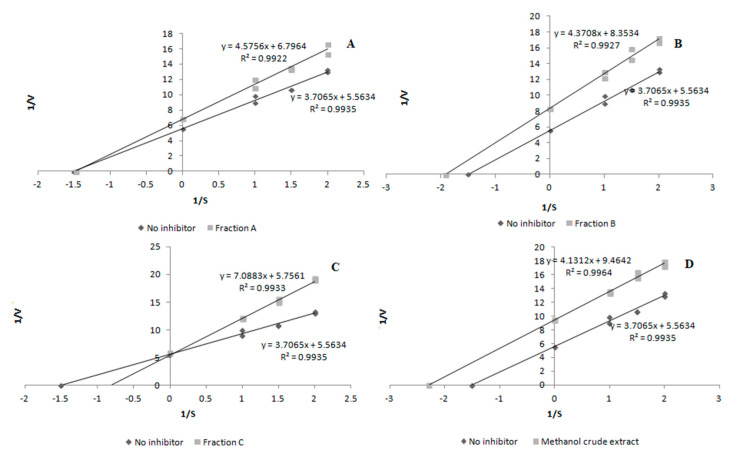
Mode of xanthine oxidase (XO) inhibition by samples from *Aerva lanata* (L.) Juss. herb. (**A**) Fraction A; (**B**) fraction B; (**C**) fraction C; (**D**) crude methanol extract. V, rate of enzymatic reaction expressed as the absorbance unit; S, substrate concentration.

The next stage of our study was to determine the mechanism of action of XO inhibitors. As presented in Figure 3, the inhibitory activity of fraction B and crude methanol extract was classified as uncompetitive, while fraction A and fraction C exhibited the non-competitive and competitive types of inhibition, respectively.

Pearson correlation coefficients and *R*^2^ coefficients were calculated to show the relationship between the DPPH^•^ radical scavenging activity, ABTS^•+^ scavenging assay, and content of phenolic acids in the *Aerva lanata* (L.) Juss. fractions.

Table 3 shows correlation coefficients between the content of phenolic acids and free radical scavenging ability. The results indicate a strong correlation between the content of a majority of the analysed acids and antioxidant activity measured with the DPPH^•^ and ABTS^•+^ methods. The highest correlation coefficients between phenolic acids and DPPH^•^ were found for rosmarinic acid (*r* = 0.9998), salicylic acid (*r* = 0.9986), syringic acid (*r* = 0.9956), and protocatechuic acid (*r* = 0.9868). Considerable results were also obtained for sinapic acid (*r* = 0.9594) and gallic acid (*r* = 0.9249).The same phenolic acids as in the case of DPPH^•^ influenced the activity of ABTS^•+^; the highest correlation was obtained for rosmarinic acid (*r* = 0.9996), salicylic acid (*r* = 0.9990), syringic acid (*r* = 0.9949), protocatechuic acid (*r* = 0.9880), and sinapic acid (*r* = 0.9616). Gallic acid (*r* = 0.9279) and gentistic acid (*r* = 0.9137) exerted a relatively high effect on antioxidant activity. In most cases, these acids had a high effect on both types of anti-radical activity.

The correlation coefficients indicate that the total content of phenolic acids in the dry fraction had a significant impact on DPPH^•^ radical scavenging activity (*r* = 0.5667) and ABTS^•+^ scavenging activity (*r* = 0.5779).

The antioxidant activities are related to the structures of phenolic compounds [46,47]. Generally, antioxidant activity depends on the number and positions of hydroxyl groups and other substituents [48,49]. Synergism ensures a favourable effect of the use of two antioxidants simultaneously, one reinforcing the effect of the other. A mixture of two different phenolic acids can have a synergistic effect, resulting in a higher efficiency than that of single antioxidants [50]. As in the case of antioxidant activity, the structure of the phenolic compound has the greatest impact on anti-diabetic activity. The total hydroxyl groups, hydroxyl configuration, double bond, and C-4 ketonic functional group are the basic characteristics of the biological activity of phenolic compounds determining their anti-diabetic properties [26].

### 2.3. Anti-Diabetic Activity

The anti-diabetic activity of the crude methanol extract and dry fractions obtained from *Aerva lanata* (L.) Juss. was determined, and the results of this research are shown in Table 4. Among the fractions, fraction B (EC_50_ = 0.30 mg dry extract/mL) showed the strongest α-glucosidase inhibitory activity, followed by fraction C (EC_50_ = 0.71 mg/mL). Interestingly, approximately 2-fold higher values of the inhibitory effects were recorded in comparison with the standard α-glucosidase inhibitor, i.e., acarbose (1.39 mg/mL). The results of α-amylase inhibition by the fractions were contrary to those of α-glucosidase inhibition. The maximum α-amylase inhibitory effect was observed in fraction A (EC_50_ = 3.51 mg/mL), but it was weaker than in the case of acarbose. This was followed by fraction C (EC_50_ = 5.46 mg/mL) and fraction B (EC_50_ = 7.46 mg/mL).

Type 2 diabetes develops due to tissue resistance to insulin and dysfunction of islet of Langerhans β-cells. The state of insulin resistance is related to oxidative stress in both β-cells and other tissues targeted by insulin. Oxidative stress contributes to the loss of β-cell function and to the aggravation of insulin resistance. In diabetics, the level of oxidative damage increases as the disease progresses. Diabetes is accompanied by reduced activity of antioxidant enzymes [51]. Various sources of antioxidant phenolic acids have been found to reduce the risk of diabetes. Most phenolic acids have exhibited significant anti-diabetic activity [29].

Fraction A (fraction of free phenolic acids) displayed the lowest α-glucosidase inhibitory capacity compared with the other fractions; however, it was higher than that of the acarbose standard and the crude methanol extract. Fraction C (fraction of phenolic acids obtained after alkaline hydrolysis) had 2-fold higher α-glucosidase activity than acarbose, while fraction B (fraction of phenolic acids obtained after acidic hydrolysis) was approximately 4-fold more potent (Table 3). The crude methanol extract had the lowest activity against α-glucosidase. In turn, all fractions exhibited much lower α-amylase activity than acarbose. Fraction A caused the strongest inhibition of α-amylase, which was reflected in the lowest EC_50_, compared with that determined for the other fractions.

One of the important therapeutic strategies to decrease postprandial hyperglycaemia is to retard the absorption of glucose through inhibition of carbohydrate hydrolysingenzymes. α-Glucosidase is one of the enzymes involved in the release of glucose from starch for intestinal glucose absorption. The inhibition of this enzyme decreases blood glucose levels. Acarbose is an anti-diabetic drug inhibiting α-glucosidase activities. The major side effects of acarbose include diarrhoea and flatulence as a result of prolonged inhibition of starch hydrolysis [52].

Excessive inhibition of pancreatic α-amylase may result in abnormal bacterial fermentation of undigested carbohydrates in the colon, hypoglycaemia, and abdominal distention; therefore, mild α-amylase inhibition activity is useful [53,54]. Fraction B had the best anti-diabetic properties due to its mild inhibition of α-amylase (EC_50_ = 7.46 mg/mL) and strong inhibition of α-glucosidase (EC_50_ = 0.30 mg/mL). This is confirmed by available studies showing that plant-origin anti-diabetic drugs exhibit stronger inhibition activity against α-glucosidase and a lower inhibitory effect against α-amylase activity with minimal side effects [53].

In summary, although dry fraction B did not exhibit the highest content of total phenolic acids, it had the highest anti-diabetic potential and antioxidant activity. The acidic hydrolysis resulted in the largest release of syringic (5.580 mg per g dry fraction), vanillic (4.160 mg per g dry fraction), gentistic (2.760 mg per g dry fraction), salicylic (1.868 mg per g dry fraction), and protocatechuic acids (1.130 mg per g dry fraction), which indicates their greatest impact on the types of activity studied.

There are other studies on the anti-diabetic and antioxidant properties of *Aerva lanata* (L.) Juss. extracts. In their study, Akanji et al. evaluated the inhibitory effects of aqueous, ethanolic, and aqueous-ethanolic extracts of *A. lanata* (L.) Juss. leaves on the activities of diabetes-related enzymes and antioxidant potential. The aqueous-ethanolic extract was found to inhibit α-amylase (IC_50_ = 2.42 mg/mL) and α-glucosidase (IC_50_ = 0.23 mg/mL). The aqueous-ethanolic and aqueous extracts displayed the best DPPH^•^ and ABTS^•+^ radical-scavenging ability [24]. Additionally, in another study, ethanolic extract and ethyl acetate extract caused inhibition of yeast α-glucosidase (IC_50_ = 81.76 μg/mL and 108.23 μg/mL, respectively). Both extracts also inhibited rat intestinal α-glucosidase (IC_50_ value of ethanolic extract and ethyl acetate extract: 108.7 and 208.04 μg/mL, respectively) [23].

Gallic acid and acarbose in various combinations inhibited the activity of α-amylase and α-glucosidase in in vitro studies. Noteworthy, 100% acarbose had more potent inhibitory activity than 100% gallic acid [52]. Furthermore, in vitro and in vivo studies revealed that gallic acid (and tannic acid) contributed to improvement of the therapeutic properties of acarbose by enhancing its antioxidant properties. This indicates that phenolic acid-rich diets have antioxidant properties and exert hypoglycaemic effects. It is possible to achieve significant antihyperglycaemic effects by a combination of phenolic acids with a reduced dose of acarbose and thus minimize the side effects of its action [55].

Table 5 presents correlation coefficients between the content of phenolic acids and α-glucosidase and α-amylase inhibitory activities. In the case of α-glucosidase, the presence and content of syringic (*r* = −0.9948), rosmarinic (*r* = −0.9848), gentistic (*r* = −0.9767), salicylic (*r* = −0.9700), vanillic (*r* = −0.9454), and protocatechuic (*r* = −0.9370) acids havethe greatest effect on the α-glucosidase inhibitory capacity. In addition, some of these acids exhibit a high correlation with α-amylase inhibiting activity: rosmarinic acid (*r* = 0.9999), salicylic acid (*r* = 0.9985), syringic acid (*r* = 0.9960), and protocatechuic acid (*r* = 0.9863). Isoferulic, p-coumaric, ferulic, and caffeic acids show a very low correlation indicating that their content has a low impact on both α-glucosidase and α-amylase inhibition. The results show the highest content of phenolic acids in dry fraction C, whereas dry fraction B exhibits the lowest activity towards α-amylase and the highest activity towards α-glucosidase, DPPH^•^, and ABTS^•+^.

The correlation coefficients indicate that the total content of phenolic acids in the dry fraction had a lower effect on α-glucosidase inhibitory activity (*r* = −0.4024) and α-amylase inhibitory activity (*r* = 0.5644). Compared withdry fractions A and C, dry fraction B contained higher amounts of syringic, rosmarinic, gentistic, salicylic, and protocatechuic acids. All these acids showed a high correlation with α-glucosidase and α-amylase inhibitory activities (*r* > 0.9).

As reported by other authors, sinapic, gallic, protocatechuic, gentistic, vanillic, and syringic acids have α-glucosidase inhibitory activity [56,57], which was also confirmed by our research. There are also many in vitro and in vivo reports on the anti-diabetic effects of gallic, protocatechuic, syringic, salicylic, ferulic, caffeic, p-coumaric, sinapic, and rosmarinic acids [29]. Studies on humans or rodents provide ample evidence showing that consumption of phenolic acids reduces the diabetes risk by regulation of the key pathway of carbohydrate metabolism and hepatic glucose homeostasis [29].

## 3. Material and Methods

### 3.1. Plant Material

Herb of *Aerva lanata* (L.) Juss. (series no. 51018) was purchased from the herb manufacturer “Ликтpaви” (Kyivs’ke Hwy, 21, Zhytomyr, Zhytomyr Oblast, Ukraine). According to the information provided by the manufacturer, the herb material was dried and packed appropriately.

### 3.2. Chemicals

All phenolic acid standards, Trolox, 2,2-diphenyl−1-picrylhydrazyl radical (DPPH^•^), 2,2′-azino-bis-3(ethylbenzthiazoline-6-sulphonic acid) (ABTS^•^^+^), soybean 15-lipooxygenase, xanthine oxidase, linoleic acid, allopurinol, quercetin, α-glucosidase, p-nitrophenyl-α-D-glucopyranoside, α-amylase, starch solution, dinitrosalicylic acid, Rochelle salt, and acarbose were provided by Sigma-Aldrich Chemical Co. (St. Louis, MO, USA). Methanol, ethanol, chloroform, diethyl ether, sodium bicarbonate, hydrogen chloride, anhydrous sodium sulphate, sodium borohydride, sulphuric acid, sodium carbonate, sodium hydroxide, and phosphate buffer were purchased from Avantor Performance Materials Poland (Gliwice, Poland). All the chemicals were of analytical grade. LC grade methanol and formic acid were purchased from J.T. Baker (Phillipsburg, NJ, USA). LC grade water was prepared using a Millipore Direct-Q3 purification system (Bedford, MA, USA).

### 3.3. Extraction Procedure and Isolation of Phenolic Acid Fractions

The extraction method applied for isolation of phenolic acids was based on the method proposed by Schmidtlein and Herrmann [58] with some modifications described previously [59,60]. Powdered herb of *Aerva lanata* (L.) Juss. (100g) was extracted three times with boiling 80% (*v*/*v*) methanol under reflux (50 min). The methanolic extracts were filtered, pooled, and evaporated to dryness under reduced pressure. The dry extract was dissolved in hot distilled water and, after cooling, placed in a refrigerator (4 °C) for 12 h. Then, the solution was centrifuged, and the supernatant was extracted with chloroform and diethyl ether successively. The diethyl ether fraction was used for isolation of free phenolic acids, while the remaining aqueous layer was divided into two parts (W1 and W2) and subjected to acid and alkaline hydrolysis to release phenolic acids from glycoside and ester linkages, respectively (Figure S1 in Supplementary Materials).

### 3.4. Isolation of Free Phenolic Acid Fraction (FA)

The diethyl ether extract was extracted with a 5% (*w*/*v*) aqueous sodium bicarbonate solution. The bicarbonate extract was acidified to pH = 2–3 with 10% (*v*/*v*) HCl and again eluted with diethyl ether. The ether extract was washed, dried with anhydrous sodium sulphate, and evaporated to obtain a free phenolic acid fraction (FA).

### 3.5. Isolation of Bound Phenolic Acid Fractions after Acidic (B) and Alkaline Hydrolysis (C)

Water extract (W1) was hydrolysed with 2M HCl (under reflux, 100 °C, 1 h). The cool extract was filtered and next extracted with diethyl ether. Then, fraction B (FB) containing phenolic acids released after acidic hydrolysis was obtained in an analogous procedure to that described for FA.

Alkaline hydrolysis was performed on the second half volume of water extract (W2). The sample was heated under reflux with 1% (*w/v*) Ba(OH)_2_ with sodium borohydride (NaBH_4_) (pH of 12–13, 100 °C, 15 min). Then, after cooling, it was acidified to pH 2–3 with H_2_SO_4_. The mixture was filtered and extracted with diethyl ether, and fraction C of phenolic acids released after alkaline hydrolysis (FC) was obtained as described above.

### 3.6. SPE Procedure

The dry residues of fractions FA, FB, and FC were weighed and dissolved in 5 mLof 30% methanol in 0.1% (*v*/*v*) HCOOH. The solutions were passed through C18 solid-phase extraction cartridges (Octadecyl C18, Sigma-Aldrich, Fine Chemicals, St. Louis, MO, USA) preconditioned with water, methanol, and 30% (*v*/*v*) methanol in 0.1% (*v*/*v*) HCOOH. The eluates were directly analysed with the LC MS/MS method.

The scheme of the process of extraction of the phenolic acid fractions is shown in Figure S1 (Figure S1 in the Supplementary Materials). 

### 3.7. LC-ESI-MS/MS Analysis

Phenolic acids were determined by reversed-phase high-performance liquid chromatography and electrospray ionization mass spectrometry (LC-ESI-MS/MS). An Agilent 1200 Series HPLC system (Agilent Technologies, Santa Clara, CA, USA) equipped with a binary gradient solvent pump, a degasser, an autosampler, and a column oven connected to a 3200 QTRAP mass spectrometer (AB Sciex, Framingham, MA, USA) were used.

The LC-ESI-MS/MS method used in this study is a modification of the method described previously by Nowacka et al. [61]. The compounds were separated at 25 °C on a Zorbax SB-C18 column (2.1 × 100 mm, 1.8µm particle size; Agilent Technologies). The injection volume was 5 µL. Gradient elution was applied using water containing 0.1% (*v*/*v*) HCOOH (A) and acetonitrile with 0.1% (*v*/*v*) HCOOH (B). The flow rate was 300 µL/min and the gradient was as follows: 0–2 min, 18% B; 3–4 min, 25% B; 5–6 min, 35% B; 8–12 min, 60% B; 14–16 min, 75% B. The total run time was 28 min.

The ESI-MS worked in the negative ion mode, and the parameters were optimized: capillary temperature 450 °C, curtain gas at 30 psi, nebulizer gas at 50 psi, source voltage −4500 V. Nitrogen was used as the nebulizer and collision gas.

The quantitative analysis of compounds was performed using multiple reaction monitoring (MRM), and Analyst 1.5 software (AB Sciex, Foster City, CA, USA) was used for data acquisition and analysis.

Triplicate injections were made for each standard solution and samples. The limits of detection (LOD) and quantification (LOQ) for all compounds were determined at a signal-to-noise ratio of 3:1 and 10:1, respectively. The identified analytes were quantified based on theirpeak area and comparison with the calibration curve for corresponding standards. The linearity range for the calibration curve was specified. Detailed conditions of the LC-MS analysis are given in Tables S1 and S2 (in the Supplementary Materials).

### 3.8. Antioxidant Activity Analysis

The antioxidant assay was assessed with the DPPH^•^ (2,2-diphenyl-1-picrylhydrazyl) method with some modifications [62,63]. The mixtures were shaken and incubated at 28 °C for 30 min. Absorbance was measured at 517 nm using the Infinite Pro 200F microplate reader (Tecan Group Ltd., Männedorf, Switzerland). Methanol instead of the samplewas the blank. The inhibition curves were prepared and EC_50_ values, defined as the amount of the antioxidant necessary to decrease the initial DPPH^•^ concentration by 50%, were determined. The results were expressed as Trolox equivalent antioxidant capacity (TE) (mM of Trolox per g fraction/extract) based on their EC_50_ values.

The antiradical activity was determined using an improved ABTS^•+^ decolourization assay with modifications [64]. The 96-well microplate technique was used to determine the EC_50_ value of the samples. Aliquots of 180 μL of an ABTS^•+^ solution were mixed with 20 Μl of the samples diluted to various concentrations. The samples were shaken and incubated at 28 °C. Absorbance was measured after 6 min incubation at 734 nm using the Infinite Pro 200F microplate reader (Tecan Group Ltd., Männedorf, Switzerland). Methanol instead of the sample was the blank. The results of the antioxidant activity are expressed as Trolox equivalent antioxidant capacity (TE) (mM of Trolox per g fraction/extract) based on their EC_50_ values.

### 3.9. Anti-Inflammatory Activity

#### 3.9.1. Inhibition of Xanthine Oxidase Activity (XO)

The inhibition of XO with xanthine as a substrate was measured spectrophotometrically based on Sweeney et al. [65] with slight modification. The enzyme activity was determined at 30 °C by measuring the increase in absorbance at 295 nm over a 2min period using a microplate reader (Epoch 2 Microplate Spectrophotometer, BioTek Instruments). Ethanol instead of the sample was the control. Allopurinol was applied as inhibitor control.

The inhibitory activity (depending on the sample) was expressed as an EC_50_-extract concentration providing 50% of activity based on a dose-dependent mode of action. The mode of inhibition on the enzyme was shown using a Lineweaver–Burk plot.

#### 3.9.2. Inhibition of Lipoxygenase Activity (LOX)

Inhibition of LOX (15-lipoxygenase) with linoleic acid as a substrate was measured as in Axelrod et al. [66] with some modifications. The mixture contained 240 µL of phosphate buffer (1/15 M, pH 7.5), 10 μL of *Aerva lanata* (L.) Juss. sample, 10 μL of a LOX solution, and 40 μL of 2.5 mM linoleic acid. The enzyme was determinate at 30 °C measuring the increase in absorbance at 234 nm over a 2 min period. Ethanol instead of the sample was the control. Quercetin was applied as inhibitor control.

The inhibitory activity was expressed as an EC_50_-extract concentration providing 50% of activity based on a dose-dependent mode of action. The mode of inhibition on the enzyme was shown using a Lineweaver–Burk plot.

### 3.10. Anti-Diabetic Activity

#### 3.10.1. α-Glucosidase Inhibition Assay

The α-glucosidase inhibitory activity of the extracts was determined with the method described by Telagari and Hullati [67]. A mixture containing 10 µL of α-glucosidase (from *Saccharomyces cerevisiae*) (1 U/mL; in phosphate buffer, 0.1 M, pH 6.8), 50 µL of 0.1 M phosphate buffer (pH 6.8), and 20 µL of the tested sample at various concentrations was incubated in 96-well microplates at 37 °C for 15 min. After pre-incubation, the enzymatic reaction was initiated by addition of 20 µL of a 5 mM p-nitrophenyl-α-D-glucopyranoside solution in 0.1 M phosphate buffer (pH 6.8), and the reaction mixture was incubated for another 20 min at 37 °C. The reaction was terminated by addition of 50 µL of a Na_2_CO_3_ solution (0.1 M). Absorbance was determined at 405 nm using an ELISA microplate reader. The reaction system without plant extracts was used as a control, and the system without α-glucosidase was used as a blank for correction of the background absorbance. The α-glucosidase inhibitory activity was expressed as the EC_50_ value according to the percentage inhibition and calculated with the following formula:

% inhibition = (1 − sample absorbance/control absorbance) × 100

#### 3.10.2. α-Amylase Inhibition Assay

The α-amylase inhibition assay used in this study was previously described by Abirami et al. [68]. The sample (100 µL) was mixed with 100 µL of 0.02 M sodium phosphate buffer (pH 6.9) and 100 µL of an α-amylase solution (4.5 U/mL/min) and pre-incubated at 25 °C for 10 min. Then, 100 µL of a 1% starch solution was added and incubated at 25 °C for 30 min. The reaction was stopped by addition of 1.0 mL of dinitrosalicylic acid reagent (1 g of 3,5-dinitrosalicylic acid in a solution containing 20 mL of 2 M NaOH, 50 mL of distilled water, and 30 g of Rochelle salt; the contents were dissolved, and the volume was made up to 100 mL with distilled water). The test tubes were then incubated in a boiling water bath for 5 min and then cooled to room temperature. The reaction mixture was then diluted 10 times with distilled water and the absorbance was measured at 540 nm. The readings were compared with the control (the sample was replaced by buffer),and α-amylase inhibition activity (%) was calculated. The α-amylase inhibitory activity was expressed as the EC_50_ value according to the percentage inhibition and calculated with the following equation formula:

% inhibition = (1 − sample absorbance/control absorbance) × 100

## 4. Statistical Analysis

All tests were performed in triplicate, and the results areexpressed as a mean ± standard deviation (SD). Moreover, Pearson correlation coefficients and R^2^ coefficients were calculated to show the relationship between antioxidant activity measured with the DPPH^•^ and ABTS^•+^ method and α-glucosidase inhibitory activity of the extracts from *Aerva lanata* (L.) Juss.

A one-way ANOVA test followed by Tukey’s post hoc test was used for statistical analysis of the differences between the data. Significance was assumed at *p* < 0.05 and *p* < 0.001.

Statistical analyses were carried out using STATISTICA 10.0 (StatSoft Poland, Cracow, Poland).

## 5. Conclusions

Herb of *Aerva lanata* (L.) Juss. is widely used in folk medicine due to its multidirectional biological activity. In this work, we indicate for the first time the relationship between the presence of phenolic acids and the anti-diabetic and antioxidant activity of this plant material. The rapid, simple, accurate, and specific LC-ESI-MS/MS method was used for quantitative estimation of phenolic acid fractions. Fraction FB containing PA liberated after acidic hydrolysis showed the greatest antioxidant and anti-diabetic activity. In addition, the phenolic acid content in the dry fraction was approximately 1000 times higher than that in the dry extracts. The results presented in this paper indicate for the first time that the anti-diabetic and antioxidant activity of the fractions from *A. lanata* (L.) Juss. are closely related to the quantitative and qualitative composition of phenolic acids present in the fractions. The correlation results indicate that phenolic acids had a significant effect on the antioxidant and potential anti-diabetic properties of the fractions. The present research confirms earlier reports on the herb of *Aerva lanata* (L.) Juss. and shows that the PA composition of FB is especially interesting due to the strong antioxidant and anti-diabetic activity.

Our in vitro study revealed promising anti-inflammatory, antioxidant, and anti-diabetic activities based on several mechanisms of action. Since one of the diabetes preventive strategies is a diet with polyphenol-rich products, the search for natural agents containing these compounds seems important.

## Figures and Tables

**Table 1:** Content of phenolic acids in fractions A, B, and C obtained from *Aerva lanata* (L.) Juss. herb (mg per g dry fraction).Values are presented as mean ± standard deviation (*n* = 9).

	Fraction A	Fraction B	Fraction C
Gallic acid	0.003 ± 0.000	0.238 ± 0.006	0.203 ± 0.004
Protocatechuic acid	0.459 ± 0.009	1.130 ± 0.001	0.887 ± 0.007
Caffeic acid	0.241 ± 0.010	1.996 ± 0.057	5.190 ± 0.009
Syringic acid	0.275 ± 0.001	5.580 ± 0.009	2.480 ± 0.008
4-hydroxybenzoic acid	1.370 ±0.010	1.520 ± 0.060	1.612 ± 0.010
Vanillic acid	2.137 ± 0.009	4.160 ± 0.008	2.120 ± 0.007
Gentisic acid	0.028 ± 0.001	2.760 ± 0.001	0.351 ± 0.001
Sinapic acid	0.060 ± 0.000	2.660 ± 0.002	2.010 ± 0.001
p-Coumaric acid	2.320 ± 0.003	3.570 ± 0.002	9.410 ± 0.024
Ferulic acid	1.886 ± 0.012	4.810 ± 0.004	11.160 ± 0.250
Rosmarinic acid	0.015 ± 0.000	0.086 ± 0.000	0.049 ± 0.000
Isoferulic acid	1.486 ± 0.015	5.120 ± 0.235	17.140 ± 0.678
Salicylic acid	0.569 ± 0.020	1.868 ± 0.009	1.271 ± 0.001
Total phenolic acids	10.849	35.498	53.883

**Table 2:** Results of the biological assays of phenolic acid fractions from *Aerva lanata* (L.) Juss. The results of the DPPH and ABTS cation radical assays are expressed as mM TE (Trolox equivalents)·g^−1^ dry weight of fraction/extract. The results of XO and LOX activities are expressed as EC_50_ mg·mL^−1^ dry weight of fraction/extract.

	Antioxidant Activity		Activity against	
	DPPH^•^	ABTS^•+^	Xanthine Oxidase	Lipoxygenase
	TE (mM Trolox·g^−1^ Dry Fraction/Extract)		EC_50_ (mg·mL^−1^ Dry Fraction/Extract)	
Fraction A	0.49 ^a^ ± 0.02	0.57 ^a^* ± 0.03	2.63 ^a^ ± 0.76	4.16 ^a^ ± 0.10
Fraction B	2.85 ^b^ ± 0.03	2.86 ^b^ ± 0.22	1.88 ^b^ ± 0.34	1.77 ^b^ ± 0.03
Fraction C	1.66 ^c^ ± 0.02	1.74 ^c^ ± 0.02	4.15 ^c^ ± 0.23	3.42 ^c^± 0.03
Crude methanol extract	0.29 ^d^ ± 0.01	0.22 ^d^* ± 0.01	2.38 ^d^ ± 0.45	3.76 ^d^ ± 0.07

Values are presented in mean ± standard deviation (*n* = 3) and evaluated by one-way ANOVA (post hoc test: Tukey). Different superscript letters (^a−d^) in the same column denote significant differences at *p* < 0.001 (* *p* < 0.05).

**Table 3:** Pearson correlation coefficients *r*(X,Y) and coefficients of determination (*R*^2^) showing a relationship between phenolic acids and DPPH^•^ and ABTS^•+^ for *Aerva lanata* (L.) Juss. fractions.

Phenolic Acid	DPPH^•^		ABTS^•+^	
	*r*(X,Y)	*R* ^2^	*r*(X,Y)	*R* ^2^
Gallic acid	0.9249	0.8554	0.9279	0.8611
Protocatechuic acid	0.9868	0.9737	0.9880	0.9762
Caffeic acid	0.3451	0.1191	0.3527	0.1244
Syringic acid	0.9958	0.9915	0.9949	0.9899
4-OH-benozoic acid	0.6101	0.3722	0.6165	0.3801
Vanillic acid	0.8648	0.7480	0.8607	0.7408
Gentistic acid	0.9170	0.8410	0.9137	0.8348
Sinapic acid	0.9594	0.9204	0.9616	0.9247
p-Coumaric acid	0.1603	0.0257	0.1683	0.0283
Ferulic acid	0.3037	0.0922	0.3114	0.0969
Rosmarinic acid	0.9998	0.9996	0.9996	0.9992
Isoferulic acid	0.2170	0.0471	0.2249	0.0505
Salicylic acid	0.9987	0.9973	0.9990	0.9981
Total phenolic acids	0.5667	0.3212	0.5779	0.3340

**Table 4:** EC_50_ values of in vitro α-glucosidase and α-amylase inhibition by the crude methanol extract and fractions from *Aerva lanata* (L.) Juss.

Sample	α-Glucosidase Inhibitory Effect	α-Amylase Inhibitory Effects
	EC_50_ (mg mL^−1^ Dry Extract)	
Fraction A	0.90 ^a^* ± 0.07	3.51 ^a^ ± 0.02
Fraction B	0.30 ^b^ ± 0.04	7.46 ^b^ ± 0.11
Fraction C	0.71 ^a^ ± 0.08	5.46 ^c^ ± 0.03
Crude methanol extract	5.52 ^c^ ± 0.01	NA
Acarbose	1.39 ^d^* ± 0.01	0.01 ^d^ ± 0.03

Values are presented as mean ± standard deviation (*n* = 3) and evaluated by one-way ANOVA (post hoc test: Tukey). Different superscript letters ^(a–d)^ in the same column denote significant differences at *p* < 0.001 (* *p* < 0.05). Abbreviations: NA, no activity.

**Table 5:** Pearson correlation coefficients *r* (X,Y) and coefficients of determination (*R*^2^) showing a relationship between phenolic acids and α-glucosidase inhibition for *Aerva lanata* (L.) Juss. fractions.

Phenolic Acid	α-Glucosidase Inhibition		α-Amylase Inhibition	
	*r* (X,Y)	*R* ^2^	*r* (X,Y)	*R* ^2^
Gallic acid	−0.8343	0.6960	0.9238	0.8534
Protocatechuic acid	−0.9370	0.8781	0.9863	0.9728
Caffeic acid	−0.1578	0.0249	0.3425	0.1173
Syringic acid	−0.9948	0.9896	0.9960	0.9920
4-OH-benozoic acid	−0.4460	0.1989	0.6079	0.3695
Vanillic acid	−0.9453	0.8937	0.8663	0.7504
Gentistic acid	−0.9766	0.9538	0.9181	0.8430
Sinapic acid	−0.8870	0.7869	0.9586	0.9189
p-Coumaric acid	0.0328	0.0010	0.1575	0.0248
Ferulic acid	−0.1144	0.0130	0.3010	0.0906
Rosmarinic acid	−0.9848	0.9698	0.9999	0.9997
Isoferulic acid	−0.0248	0.0006	0.2142	0.0459
Salicylic acid	−0.9700	0.9409	0.9985	0.9970
Total phenolic acids	−0.4024	0.1619	0.5644	0.3185

## Supplementary Materials

Figure S1: Scheme of extraction of phenolic acids from fractions obtained from *Aerva lanata* (L.) Juss., Table S1: LC-ESI-MS/MS analytical results of phenolic acids investigated in samples. Compounds were confirmed by comparison with authentic standards, Table S2: Limit of detection (LOD), limit of quantification (LOQ), and calibration curve parameters for phenolic acids.

## Sample Availability

Samples of the compounds are available from the authors.
