# Supplementary material for: Antioxidant, Anti-Inflammatory, and Anti-Diabetic Activity of Phenolic Acids Fractions Obtained from Aerva lanata (L.) Juss
Source: Molecules. 2021 Jun 8;26(12):3486. doi: 10.3390/molecules26123486 (PMC8228310; doi:10.3390/molecules26123486)
Supplement: Supplementary file 1 [file molecules-26-03486-s001.zip › molecules-1235721-supplementary.pdf]

Supplementary materials

Antioxidant, anti-inflammatory, and anti-diabetic activity of phenolic acids fractions obtained from *Aerva lanata* (L.) Juss.

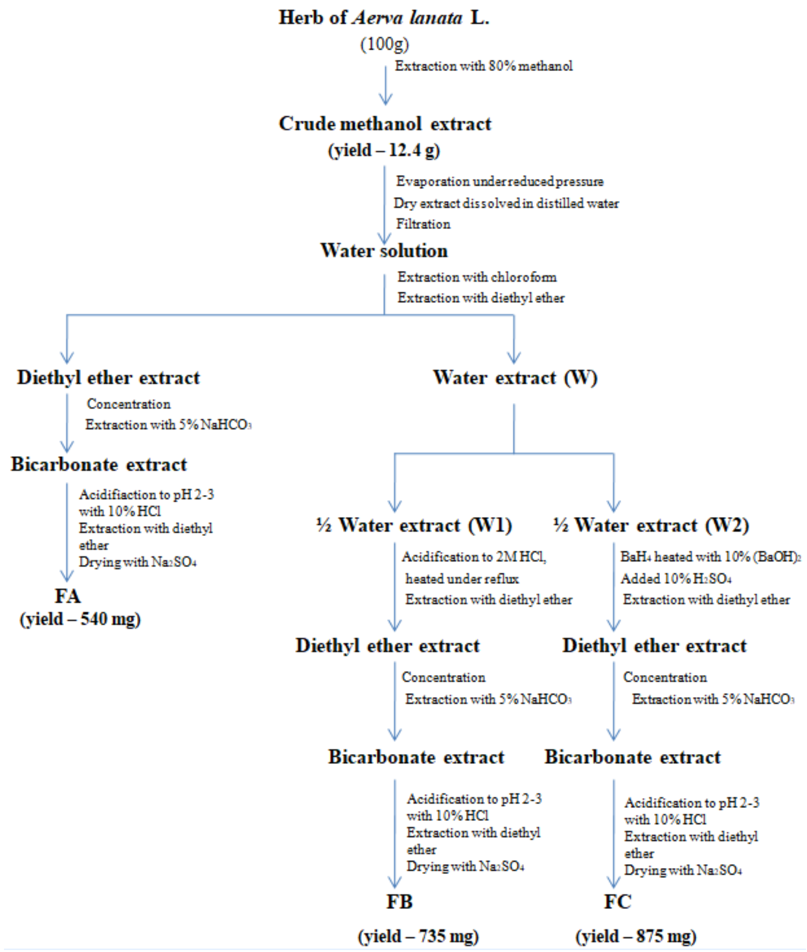

Figure S1: Scheme of extraction of phenolic acids from fractions obtained from *Aerva lanata* L.

Table S1: LC-ESI-MS/MS analytical results of phenolic acids investigated in samples. Compounds were confirmed by comparison with authentic standards

Compound	Retention time [min]	$[M-H]^-$ [m/z]	Fragment ions [m/z]	Collision energy [eV]
Gallic acid	5.00	168.7	78.9<br>124.9	-36<br>-14
Homogentisic acid	5.27	166.8	123<br>108	-12<br>-36
α-resorcylic acid	5.82	152.8	109<br>64.9	-12<br>-20
Protocatechuic acid	5.85	152.9	80.9<br>107.8	-26<br>-38

Caffeic acid	6.70	178.7	88.9<br>134.9	-46<br>-16
Syringic acid	7.14	196.9	122.8<br>181.9	-24<br>-12
4-hydroxybenzoic acid	7.27	136.8	92.9	-18
Vanillic acid	7.46	166.8	107.9<br>123	-18<br>-12
Gentisic acid	7.72	352.9	80<br>96.9	-110<br>-52
γ-Resorcylic acid	7.90	152.8	108.9<br>65	-14<br>-30
3-hydroxybenzoic acid	8.08	136.9	93<br>75	-16<br>-48
β-Resorcylic acid	8.92	152.8	108.9<br>65	-12<br>-24
Sinapic acid	9.19	222.8	121<br>148.9	-36<br>-20
p-Coumaric acid	9.28	162.8	93<br>119	-44<br>-14
Ferulic acid	9.84	192.8	133.9<br>177.9	-16<br>-12
Rosmarinic acid	10.18	358.7	132.6<br>160.8	-44<br>-20
Isoferulic acid	10.49	192.8	133.9<br>177.9	-16<br>-12
m-Coumaric acid	10.69	162.8	91<br>119	-36<br>-14
Veratric acid	10.80	180.7	121.9<br>136.9	-18<br>-12
o-Coumaric acid	11.87	162.8	119<br>93	-14<br>-46
Salicylic acid	14.16	136.9	75<br>93	-48<br>-16

Table S2: Limit of detection (LOD), limit of quantification (LOQ), and calibration curve parameters for phenolic acids

**Compound**	**LOD [ng/mL]**	**LOQ [ng/mL]**	**R^2**	**Linearity range [ng/mL]**
Gallic acid	47.5	95	0.9980	190-3300
Homogentisic acid	34	68	0.9995	136-11100
Protocatechuic acid	17	34	0.9992	68-3470
α-Resorcylic acid	174	347	0.9990	347-3470
Caffeic acid	40	160	0.9990	320-3500
Syringic acid	166	666	0.9985	666-11100

4-hydroxybenzoic acid	17	34.7	0.9984	104.1-3470
Vanillic acid	125	250	0.9987	500-33000
Gentisic acid	1.6	3.3	0.9991	3.3-330
γ-Resorcylic acid	40	200	0.9982	200-7000
3-hydroxybenzoic acid	84	167	0.9985	334-6670
β-Resorcylic acid	50	100	0.9992	200-2000
Sinapic acid	17.4	69.4	0.9991	138.8-3470
p-Coumaric acid	7.3	18.1	0.9991	36.2-1820
Ferulic acid	17.4	34.7	0.9991	69.4-11600
Rosmarinic acid	7.1	17.9	0.9990	35.8-7140
Isoferulic acid	19	686	0.9985	686-11400
m-Coumaric acid	17	136	0.9987	174-17000
Veratric acid	1570	3140	0.9985	3140-31400
o-Coumaric acid	7.3	21.9	0.9991	21.9-1820
Salicylic acid	8.3	16.5	0.9985	16.5-1650
